# Healthy Lifestyle Factors, Cancer Family History, and Gastric Cancer Risk: A Population-Based Case-Control Study in China

**DOI:** 10.3389/fnut.2021.774530

**Published:** 2021-12-22

**Authors:** Jinyu Man, Yingchun Ni, Xiaorong Yang, Tongchao Zhang, Ziyu Yuan, Hui Chen, Xingdong Chen, Ming Lu, Weimin Ye

**Affiliations:** ^1^Department of Epidemiology and Health Statistics, School of Public Health, Cheeloo College of Medicine, Shandong University, Jinan, China; ^2^Clinical Epidemiology Unit, Qilu Hospital of Shandong University, Jinan, China; ^3^Clinical Research Center, Qilu Hospital of Shandong University, Jinan, China; ^4^State Key Laboratory of Genetic Engineering, Collaborative Innovation Center for Genetics and Development, School of Life Sciences, Fudan University, Shanghai, China; ^5^Fudan University Taizhou Institute of Health Sciences, Taizhou, China; ^6^Human Phenome Institute, Fudan University, Shanghai, China; ^7^Department of Medical Epidemiology and Biostatistics, Karolinska Institutet, Stockholm, Sweden; ^8^Department of Epidemiology and Health Statistics & Key Laboratory of Ministry of Education for Gastrointestinal Cancer, Fujian Medical University, Fuzhou, China

**Keywords:** family history, gastric cancer, case-control study, lifestyle factor, food storage

## Abstract

**Background:** We aimed to explore the relationship between lifestyle factors, cancer family history, and gastric cancer risk.

**Methods:** We examined the association between lifestyle factors, cancer family history, and gastric cancer risk based on a population-based case-control study in Taixing, China, with 870 cases and 1928 controls. A lifestyle score was constructed considering body shape, smoking, alcohol drinking, tooth brushing habit, and food storage method. Unconditional logistic regression models were used to calculate odd ratios (ORs) and 95% confidence intervals (CIs).

**Results:** Compared with participants with a lifestyle score of 0, subjects with a lifestyle score of 1 (OR 0.59, 95%CI 0.43–0.83), 2 (OR 0.42, 95%CI 0.30–0.59), 3 (OR 0.29, 95%CI 0.20–0.41), 4 (OR 0.20, 95%CI 0.13–0.32), or 5 (OR 0.10, 95%CI 0.04–0.22) had a lower risk of gastric cancer (*P for trend* < 0.001). Overall, 34% of gastric cancer cases (95%CI 27–41%) can be attributed to non-compliance with ≥3 healthy lifestyle. Family history of early-onset cancer is closely related to the occurrence of gastric cancer, with an OR ranging from 1.77 to 3.27. Regardless of family history, a good lifestyle is associated with a reduced risk of gastric cancer, with an OR value between 0.38 and 0.70.

**Conclusions:** The early-onset cancer family history is closely related to the occurrence of gastric cancer and a good lifestyle is associated with a reduced risk of gastric cancer regardless of family history. Our results provide a basis for identifying and providing behavior guidance of high-risk groups of gastric cancer.

## Introduction

Gastric cancer (GC) was the fifth most common cancer in the world in 2020 (1), and more than 40% of new cases and deaths from GC occurred in China (2). Family history is closely related to disease and is widely used to identify high-risk groups of many diseases, including GC (3–5). However, not all people with a family history of malignancy will get GC, which suggests that environmental and lifestyle factors play an important role in the occurrence of GC.

A number of large-scale cohort studies and meta-analyses have examined the relationship between smoking (6), alcohol drinking (7), poor oral hygiene habits (8), BMI and body composition (9, 10), diet (11–15), physical activity (16–18) and the risk of GC. The observed reduced GC risk associated with refrigerator usage also suggests that food storage method may be one of the GC-risk-related lifestyle factors (19). The reality that these lifestyle factors often appear together makes it important to explore their combined effects on GC risk. We assumed that people with healthier lifestyle have a lower GC risk than those with no or fewer healthy lifestyle factors, and the beneficial effects increase with the number of healthy lifestyle factors. Among people with different types of family history of malignancy, exploring the relationship between combined lifestyle factors and GC risk will provide information to develop the behavioral guidance for GC high-risk groups.

However, there were few studies on the relationship between combined lifestyle factors and the risk of GC (20–22). In a prospective cohort study, Jin et al. found that participants with a better lifestyle had a lower risk of GC. Compared with participants with high genetic risk and poor lifestyle, participants with high genetic risk and good lifestyle have a lower risk of GC (21). Family history reflects the common genetic background and common environmental exposure, so the genetic risk represented by the polygenic risk score does not fully represent the family history. Therefore, we still need to explore the relationship between a variety of lifestyle factors and the risk of GC among people with different types of family history of malignancy.

Taixing is one of the areas with high incidence of GC in China. Since 2010, a population-based case-control study aiming at exploring the etiology of upper gastrointestinal cancers has been carried out in Taixing, China. Based on the data collected in this project, we explored the relationship between combined lifestyle factors, family history of cancer, and GC risk, in order to provide bases for the prevention and control of GC.

## Materials and Methods

### Study Design and Setting

We have previously reported the study design and participant recruitment process in detail (23). In short, we conducted a population-based case-control study in Taixing from October 2010 to September 2013. In order to collect the newly diagnosed GC cases, we recruited cases from the endoscopy units in the four largest local hospitals. Potential missing cases were additionally identified by comparing our case list with the records of the local Cancer Registry. The GC cases included in our study were approximately 75% of the estimated new GC cases during the study period and were a good representative of the local GC patients. The potential controls were randomly selected from the local Population Registry, which provides the basic information of people living in Taixing. Trained staff used a structured electronic questionnaire to conduct face-to-face interviews with participants in the hospitals (for cases) or community (for controls and for cases identified from the local Cancer Registry). In order to reduce potential recall bias, all GC cases were interviewed before they knew their diagnoses.

### Participants

**Figure 1:**
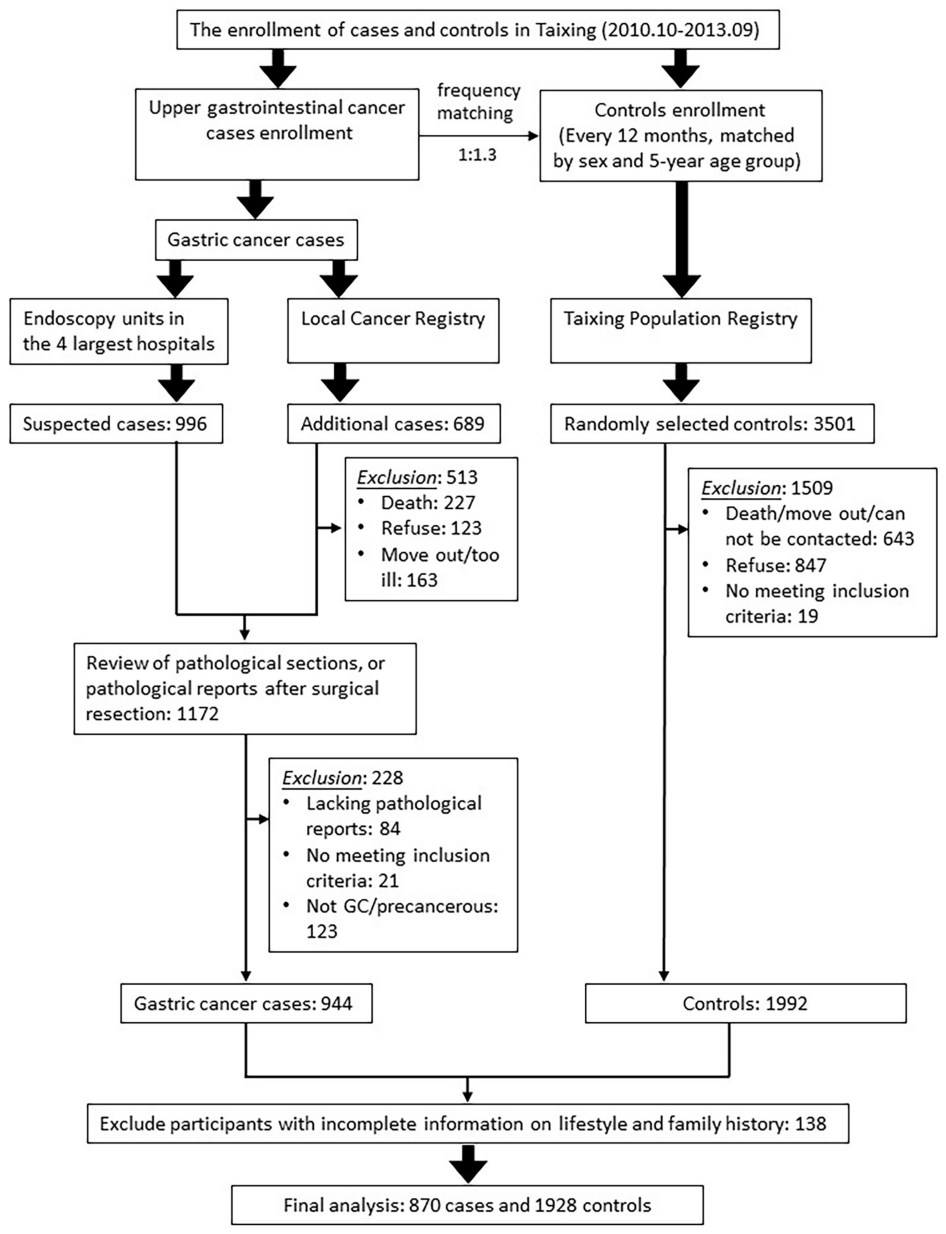
The process of inclusion and exclusion of cases and controls.

The process of inclusion and exclusion of cases and controls is shown in Figure 1. The basic inclusion criteria for participants were aged 40–85 and lived in Taixing for more than 5 years before the date of diagnosis or interview. The additional inclusion criteria for cases were: first, they were confirmed by pathological diagnosis or endoscopy; second, they have been independently verified by pathologists. The additional inclusion criteria for controls were: first, they were not diagnosed as GC; second, they were randomly selected by the frequency-matched method by gender and the 5-year age group. Based on the response rate of the controls in pilot study (response rate: 75%), we selected controls for the upper gastrointestinal cancer cases (esophageal cancer and GC) at a ratio of 1.3:1. Because the gender and age distribution of patients with esophageal cancer and GC were similar, all qualified controls were included in this analysis. We excluded those participants who were uncooperative or unable to participate in the investigation due to various reasons, such as mental illness. After excluding 138 subjects with missing lifestyle information and cancer family history information, this study finally included 870 GC cases and 1928 controls.

### Data Collection

The structured electronic questionnaire includes information on demographics (age, sex, marriage, education, family size), family wealth score, smoking and drinking status, body shape, tooth brushing, food storage method, and family history of malignancy. We defined smoking and drinking as smoking at least one cigarette every 1–3 days for 6 months and consuming alcoholic beverages at least once a week for 6 months, respectively (24). The participants were divided into never smoking/drinking and current smoker/drinker or former smoker/drinker according to their smoking or drinking history. Due to the low prevalence of obesity in Taixing city, we revised the Stunkard's Figure Rating Scale (Supplementary Figure S1), which shows the different body shapes of males and females from extremely thin (body shape 1) to extremely fat (body shape 7 for males and body shape 9 for females). The trained staff showed and introduced the revised Stunkard Graphic Rating Scale briefly, and then asked the participants to choose the closest body type 10 years before the interview. Healthy body shapes were defined as body shape 3 and body shape 4 based on previous studies (23, 25). Participants were asked how many times a day they brushed their teeth and were divided into ≥1/day and ≥2/day groups according to the frequency of tooth brushing. We collected information on food storage method by asking participants to choose the option that was closest to their food storage method 10 years before interview from six options. The six options were using airtight box inside the refrigerator, open box inside the refrigerator, airtight box outside the refrigerator, open box outside the refrigerator, plastic bag, or cloth wrap. We defined using open boxes outside the refrigerator, plastic bag, and cloth wrap as a bad food storage method, and using airtight boxes inside the refrigerator, open boxes inside the refrigerator, and airtight boxes outside the refrigerator as a good food storage method. For family history of cancer, the information we collected includes the number of siblings and children, and the cancer status of their parents, siblings, and children. For those relatives who had cancer, we further collected the information about cancer type and the age of diagnosis. A positive family history of a certain type of cancer is defined as having at least one first-degree relative suffering from the cancer. A positive family history of early onset cancer was defined as having at least one first-degree relative who was diagnosed with the cancer at or before the age of 45 (26). The status of *H. pylori* infection was obtained by quantitative detection of *H. pylori* immunoglobulin G antibody using immunoblotting assay (*H. pylori* IgG Antibody Detection Kit; Syno Gene Digital Technology, Taizhou, China).

### Lifestyle Score

After reviewing the literatures, we identified some changeable lifestyle factors related to GC risk, such as smoking, alcohol drinking, oral hygiene habits, food storage method, BMI and body composition, diet, and physical activity. Because the food storage method reflected the carcinogens that might be produced by the food (such as nitrites), and physical activity variables have not been collected in our study, the lifestyle factors finally included in our analysis are smoking, alcohol drinking, toothbrushing, food storage method, and body shape. One point was assigned to participants for the following low risk lifestyle factors: appropriate body type (shape 3/4), never smoking, never drinking, brushing teeth twice a day or more, and good food storage method. We summed the points for the five lifestyle factors to get a healthy lifestyle score which ranges from 0 (least healthy) to 5 (most healthy). Because the median lifestyle score of the participants was 2, we divided the study subjects into two groups (≤2 vs. ≥3) according to their lifestyle scores.

### Statistical Analysis

Pearson chi-squared test and Wilcoxon rank-sum test were used to evaluate the difference of distribution of the demographic, lifestyle factors and family history of cancer between case and control groups. The family wealth score was calculated based on the ownership of household appliances by the multiple correspondence analysis and classified according to the quintiles among controls (27). We used unconditional logistic regression models to calculate odds ratios (ORs) and 95% confidence intervals (CIs) to evaluate the association between family history of cancer, lifestyle factors and the risk of GC. We adjusted age (continuous) and sex in model 1 and further adjusted education (illiteracy/primary school/primary high school or above), marriage (unmarried/married/divorced or widowed), family size (<=1/2–3/>3) and family wealth score (Q1–Q5) and *H. pylori* in model 2. In Model 3, we further adjusted the GC family history, smoking, drinking, toothbrushing, food storage method, and body shape, where appropriate. In addition, we also calculated the adjusted population attributable fractions (PAF) and 95% CIs to estimate the proportion of cases attributable to lifestyle factors and lack of adherence to healthy lifestyle. PAFs were estimated based on the method proposed by Bruzzi (28). This method assumes that the cases and controls are random samples from the study population, and the exposure and confounding information are unbiased. In a case-control study, the number of cases is *x* and the number of controls is *n*−*x*. For exposure *k*, there are two levels of exposure and no exposure, and the number of cases exposed to *k* is *x*_1_. After adjusting for other confounding factors, the effect of *k* is OR. The formula for PAF is:


$$\begin{array}{l} PAF=1-\frac{1}{x}\left(\frac{x_{1}}{OR}+x-x_{1}\right)\;\left(OR>1\right) \end{array}$$

which is equivalent to the formula proposed by Miettinen (29).


$$\begin{array}{l} PAF=\frac{x_{1}}{x}\left(\frac{OR-1}{OR}\right)\;\left(OR>1\right) \end{array}$$

The 95% CIs were estimated by the bootstrap method. We sampled 1000 bootstrap samples using replacement sampling, and estimated the PAF of each bootstrap sample. The 2.5th and 97.5th percentiles of PAFs of the bootstrap samples form a good approximation of the 95% confidence interval (30). Sensitivity analysis was conducted by excluding cases from the local Cancer Registry. All analyses were performed using Stata software (version 16.0). Two-sided *P*-values less than 0.05 were considered statistically significant.

## Result

Table 1 presents the basic information of cases and controls. The mean age of cases was slightly higher than that of controls, with 67.8 years for GC cases and 66.1 years for controls. Compared with controls, cases had lower education level, lower family wealth score, and were more likely to have *H. pylori* infection. There were no significant differences between cases and controls in terms of sex, marital status and family size.

**Table 1:** The basic information of cases and controls.

**Characteristics**	**Controls** ***N* = 1928**	**Cases** ***N* = 870**	***P* value**
Age (Mean ± SD)	66.1 ± 8.8	67.8 ± 8.9	**<0.001**
Sex, %			
Male	1327(68.8)	621(71.4)	0.174
Female	601(31.2)	249(28.6)	
Education			
Illiteracy	518(26.9)	272(31.3)	**0.001**
Primary school	730(37.9)	355(40.8)	
Junior high school	519(26.9)	178(20.5)	
Senior high school and above	161(8.3)	65(7.5)	
Marriage			
Unmarried	65(3.4)	25(2.9)	0.074
Married	1538(79.8)	668(76.8)	
Divorced or widowed	325(16.9)	177(20.3)	
Family size			
<=1	724(37.6)	297(34.1)	0.186
2~3	448(23.2)	205(23.6)	
>3	756(39.2)	368(42.3)	
Family wealth score			
Q1(lowest)	388(20.1)	193(22.2)	**0.012**
Q2	404(20.9)	213(24.5)	
Q3	354(18.4)	166(19.1)	
Q4	510(26.5)	208(23.9)	
Q5(highest)	272(14.1)	90(10.3)	
*H. pylori*			
Negative	663(34.4)	225(25.9)	**<0.001**
Positive	1265(65.6)	645(74.1)	

*The calculation of P value was based on Wilcoxon rank-sum test for continuous variables, and chi-squared test for categorical variables (two-sided). Significant P values are indicated in bold*.

Table 2 shows the association between lifestyle factors, lifestyle score and the risk of GC. We observed that most individual lifestyle factors were associated with a reduced GC risk in the multivariate analysis: appropriate body type (OR 0.73, 95%CI 0.61–0.86), never drinking (OR 0.79, 95%CI 0.65–0.97), brushing teeth twice a day or more (OR 0.44, 95%CI 0.35–0.54), and good food preservation methods (OR 0.58, 95%CI 0.47–0.72). A good lifestyle was associated with a reduced GC risk in a dose-response pattern (*P* for trend < 0.001). Participants with five good lifestyle factors had only one-tenth GC risk (95%CI 0.04–0.22) compared with participants without any good lifestyle factor. However, we did not observe an association between smoking and GC risk. We further explored the relationship between food storage method and the risk of GC and found that storing food in airtight containers and low temperatures were associated with reduced GC risks, with ORs of 0.53 (95%CI 0.42–0.67) and 0.70 (95%CI 0.56–0.87), respectively (Supplementary Table S1). We also found that more than 95% of females did not smoke, and more than 70% of males smoked in both cases and controls (Supplementary Table S2).

**Table 2:** The association between lifestyle factors, lifestyle score and the risk of gastric cancer.

**Lifestyle factor**	**Points**	**Description**	**Controls** ***N =* 1928**	**Case** ***N =* 870**	**Gastric cancer**		
					**OR(95% CI)^**1**^**	**OR(95% CI)^**2**^**	**OR(95% CI)^**3**^**
Body shape	0	Unhealthy shape: shape 1/2/5/6/7/8/9	672(34.9)	364(41.8)	Ref.	Ref.	Ref.
	1	Healthy shape: shape 3/4	1256(65.1)	506(58.2)	**0.75(0.63,0.88)**	**0.75(0.64,0.89)**	**0.73(0.61,0.86)**
Smoking	0	Current smoker or former smoker	1056(54.8)	506(58.2)	Ref.	Ref.	Ref.
	1	Never smoking	872(45.2)	364(41.8)	0.89(0.71,1.11)	0.90(0.72,1.14)	0.95(0.75,1.20)
Alcohol drinking	0	Former or current drinker	788(48.9)	401(46.1)	Ref.	Ref.	Ref.
	1	Never drinking	1140(59.1)	469(53.9)	**0.80(0.66,0.96)**	**0.78(0.65,0.95)**	**0.79(0.65,0.97)**
Toothbrushing	0	<=1/day	1268(65.8)	724(83.2)	Ref.	Ref.	Ref.
	1	≥2/day	660(34.2)	146(16.8)	**0.40(0.33,0.49)**	**0.42(0.34,0.51)**	**0.44(0.35,0.54)**
Food storage method	0	Bad food storage methods	1310(67.9)	697(80.1)	Ref.	Ref.	Ref.
	1	Good food storage methods	618(32.1)	173(19.9)	**0.54(0.44,0.65)**	**0.55(0.45,0.68)**	**0.58(0.47,0.72)**
Lifestyle score	0		98(5.1)	96(11.0)	Ref.	Ref.	Ref.
	1		369(19.1)	232(26.7)	**0.63(0.45,0.87)**	**0.62(0.44,0.86)**	**0.59(0.43,0.83)**
	2		587(30.4)	275(31.6)	**0.44(0.32,0.60)**	**0.44(0.32,0.61)**	**0.42(0.30,0.59)**
	3		562(29.1)	200(23.0)	**0.30(0.21,0.42)**	**0.30(0.21,0.43)**	**0.29(0.20,0.41)**
	4		243(12.6)	59(6.8)	**0.20(0.13,0.30)**	**0.21(0.14,0.33)**	**0.20(0.13,0.32)**
	5		69(3.6)	8(0.9)	**0.10(0.04,0.21)**	**0.11(0.05,0.24)**	**0.10(0.04,0.22)**
*P* for trend					**<0.001**	**<0.001**	**<0.001**

1 *Model 1 adjusted for age and sex*.

2 *Model 2 adjusted for sex, age, education, marriage, family size, H. pylori, and family wealth score*.

3 *Model 3 adjusted for sex, age, education, marriage, family size, H. pylori, family wealth score, and family history of gastric cancer. The variables for calculating the lifestyle score were adjusted to each other in model 3. Significant ORs with 95% CIs are indicated in bold*.

Table 3 shows the PAFs according to individual and combined lifestyle factors and the risk of GC. We calculated PAFs after converting OR to > 1. The estimated PAFs attributable to non-adherence to healthy lifestyle factors were 12% (95%CI 6–16%) for body shape, 10% (95%CI 1–16%) for alcohol drinking, 47% (95%CI 38–54%) for tooth brushing, and 34% (95%CI 22–43%) for food storage method. In combination, 34% (95%CI 27–41%) of GC cases were attributable to non-adherence to healthy lifestyles (<=2 healthy lifestyle factors).

**Table 3:** PAFs according to individual and combined lifestyle factors and the risk of gastric cancer.

**Lifestyle factor**	**Gastric cancer**	
	**Proportion of cases with risk factor**	**PAF (95% CI)**
Unhealthy body shape	364(41.8%)	**12%(6%,16%)**
Smoking	506(58.2%)	3%(−12%,15%)
Alcohol drinking	401(46.1%)	**10%(1%,16%)**
Tooth brushing <= once a day	724(83.2%)	**47%(38%,54%)**
Bad food storage method	697(80.1%)	**34%(22%,43%)**
Lifestyle score <=2	603(69.3%)	**34%(27%,41%)**

*Sex, age, education, marriage, family size, H. pylori, family history of gastric cancer, and family wealth score were adjusted in the calculation of the PAF. The variables for calculating the lifestyle score were adjusted to each other. PAFs, Population attributable fractions. Significant PAFs with 95% CIs are indicated in bold*.

The relationship between family history of cancer and the risk of GC is showed in Table 4. Compared with people with no family history of cancer, people with family history of GC and other digestive system cancers showed an increased risk of GC, with OR of 2.25 (95%CI 1.76–2.88) and 1.45 (95%CI 1.17–1.79) respectively. People with family history of early-onset GC and early-onset other digestive system cancers had even higher risk of GC, with OR of 3.27 (95%CI 1.82–5.87) and 2.03 (95%CI 1.36–3.04) respectively. We did not observe a relationship between GC risk and family history of non-digestive cancers, but we observed that people with an early-onset family history of non-digestive system cancers had an increased risk of GC, with OR of 1.77 (95%CI 1.16–2.70).

**Table 4:** The association between family history of malignancy and risk of gastric cancer.

**Characteristic**	**Controls** ***N =* 1928**	**Cases** ***N =* 870**	**Gastric cancer**		
			**OR** **(95% CI)^**1**^**	**OR** **(95% CI)^**2**^**	**OR** **(95% CI)^**3**^**
Family history					
No	1055 (54.7)	391 (44.9)	Ref.	Ref.	Ref.
Yes	873 (45.3)	479 (55.1)	**1.51 (1.28,1.77)**	**1.60** **(1.36,1.89)**	**1.60 (1.36,1.90)**
Gastric cancer					
Yes	212 (11.0),	163 (18.7)	**2.19 (1.72,2.78)**	**2.21** **(1.74,2.81)**	**2.25 (1.76,2.88)**
Early onset	23 (1.2)	29 (3.3)	**3.36 (1.92,5.91)**	**3.34** **(1.89,5.88)**	**3.27 (1.82,5.87)**
Other digestive system cancer					
Yes	435 (22.6)	216 (24.8)	**1.35 (1.11,1.65)**	**1.42** **(1.16,1.75)**	**1.45 (1.17,1.79)**
Early onset	64 (3.3)	50 (5.8)	**2.03 (1.37,2.99)**	**2.06** **(1.39,3.05)**	**2.03 (1.36,3.04)**
Other cancer					
Yes	226 (11.7)	100 (11.5)	1.21 (0.93,1.58)	1.28 (0.98,1.68)	1.25 (0.95,1.65)
Early onset	65 (3.4)	43 (4.9)	**1.81 (1.21,2.72)**	**1.91** **(1.26,2.88)**	**1.77 (1.16,2.70)**

1 *Model 1 adjusted for age and sex*.

2 *Model 2 adjusted for sex, age, education, marriage, family size, H. pylori, and family wealth score*.

3 *Model 3 adjusted for sex, age, education, marriage, family size, H. pylori, family wealth, smoking, drinking, toothbrushing, food storage method, and body shape. Significant ORs with 95% CIs are indicated in bold*.

We conducted a stratified analysis for the association between lifestyle and the risk of GC. We found that participants with more healthy lifestyle factors (lifestyle score≥3) had a lower risk of GC irrespective of family history of GC (no family history of cancer: OR 0.42, 95%CI 0.32–0.57; with family history of GC: OR 0.54, 95%CI 0.33–0.91; with family history of other digestive system cancers: OR 0.70, 95%CI 0.47–1.06; with family history of non-digestive cancers: OR 0.38, 95%CI 0.19–0.76, respectively) (Table 5). Formal test for heterogeneity of results across strata also revealed no significant interaction between family history of cancer and lifestyle factors (*P* for interaction: 0.396).

**Table 5:** Association between lifestyle score and the risk of gastric cancer stratified by family history of malignancy.

**Family history of malignancy**	**Lifestyle score**	**Controls** ***N =* 1928**	**Cases** ***N =* 870**	**Gastric cancer**	
				**OR** **(95% CI)^**1**^**	**OR** **(95% CI)^**2**^**
No family history	<=2	577 (30.0)	280 (32.2)	Ref.	Ref.
	≥3	478 (24.8)	111 (12.8)	**0.42** **(0.31,0.56)**	**0.42 (0.32,0.57)**
Gastric cancer	<=2	110 (5.7)	112 (12.9)	Ref.	Ref.
	≥3	102 (5.3)	51 (5.9)	**0.54** **(0.33,0.88)**	**0.54 (0.33,0.91)**
Other digestive system cancers	<=2	232 (12.0)	137 (15.7)	Ref.	Ref.
	≥3	203 (10.5)	79 (9.1)	**0.64** **(0.44,0.95)**	0.70 (0.47,1.06)
Other cancers	<=2	135 (7.0)	74 (8.5)	Ref.	Ref.
	≥3	91 (4.7)	26 (3.0)	**0.35** **(0.19,0.65)**	**0.38 (0.19,0.76)**

1 *Model 1 adjusted for age and sex*.

2 *Model 2 adjusted for sex, age, education, marriage, family size, H. pylori, and family wealth score. Significant ORs with 95% CIs are indicated in bold*.

We also conducted a sensitivity analysis by excluding cases identified only from the local Cancer Registry, and the results showed no substantial changes (Supplementary Tables S3–S5).

## Discussion

Based on a population-based case-control study conducted in Taixing, China, we explored the association between family history of cancer, combined lifestyle factors, and the risk of GC. We found that a family history of cancer was associated with an increased GC risk. The magnitude of the association was strongest for the family history of early-onset GC, followed by the family history of GC, the family history of early-onset other digestive system cancers, the family history of early-onset non-digestive system cancers, and the family history of other digestive system cancers. Regardless of the family history of cancer, a good lifestyle is associated with a lower GC risk. Our findings provide a solid foundation to guide the prevention and control of GC.

We found that a family history of GC was significantly associated with an increased risk of GC, which was consistent with the conclusions of previous studies (31, 32). In addition, we also found that family history of other digestive system cancers and family history of early-onset other cancers were associated with an increased risk of GC. Family history reflects common environmental factors, common lifestyle, and common genetic background. One important risk factor associated with GC and affecting each other among family members is *H. pylori* infection. The *H. pylori* infection rate of family members of GC patients is higher than that of the general population, and the precancerous histological changes of the gastric mucosa are more serious in these people (33, 34). In patients with *H. pylori* infection and with a family history of GC, *H. pylori* eradication therapy can reduce the risk of GC (35). Other risk factors can also explain part of the association between family history and increased risk of GC. Previous studies have also found an association between genetic background factors such as IL-17 polymorphisms (36) and cell proliferation-related genetic polymorphisms (37) and an increased GC risk.

Most of the interviewees in our study have a relatively low education level, and the memory of weight may not be as accurate as the memory of body shape. Body shape not only reflects the weight information, but also reflects the information of body composition (23). In order to reduce recall bias and incorporate body composition information into the analysis, our study used Stunkard body shape as a component of lifestyle variables (9, 38). Consistent with previous studies, our study also found that a suitable body size is associated with a lower GC risk (10, 39). Inappropriate body shapes include overweight and underweight, which are associated with high and low BMI respectively. High BMI is associated with an increased risk of gastroesophageal reflux, which is a risk factor for gastric cardia cancer (40, 41). In addition, overweight may increase the incidence of cancer, including GC, through insulin resistance, abnormalities of the IGF-I system and signaling, and other pathways (42). Some previous studies also showed that low BMI may be associated with an increased risk of GC (10, 23). Low BMI may be associated with malnutrition and low socioeconomic status, which have been linked with a higher risk of GC (41).

Previous studies have shown that toothbrushing (43) and abstinence from alcohol can reduce the risk of GC (44), and our study supported this conclusion. Poor oral hygiene habits may lead to chronic inflammation, which is related to the occurrence of GC (43). Acetaldehyde, the metabolite of alcohol in the body, is internationally recognized as a Group 1 carcinogen to humans. In addition, alcohol also promotes the occurrence of cancer by changing the absorption and metabolism of carcinogens (45). Previous studies have shown that smoking is a risk factor for GC (41). However, no association between smoking and the GC risk was found in our study, which may be related to the homogeneous smoking habits by sex (more than 75% in males and less than 5% in females).

Our results showed that not putting food in low temperature or airtight containers may be associated with an increased risk of GC, with a high PAF of 34%. Food refrigeration and vacuum preservation can delay the spoilage process of food by preventing or inhibiting the growth of microorganisms (46). In addition, lower food storage temperature will also slow down the accumulation of carcinogens and preserve the beneficial substances in food. Studies have shown that for infant plant-based canned foods, after 24 h of refrigeration and storage at room temperature, the nitrate content increased by an average of 7 and 13%, and after 48 h of storage, the nitrate content increased by 15 and 29%, respectively (47). Higher temperatures will accelerate the aging process of fruits and vegetables and reduce the content of beneficial substances, such as carotenoids (48). These pieces of evidence strongly support our view.

In our study, compared with the people with the unhealthiest lifestyle (0 points), the risk of GC in the people with the healthiest lifestyle (5 points) was only one tenth, and this risk reduction was more eminent than that reported in previous studies (20–22, 49). The difference in the composition of lifestyle scores and the population may explain this observed heterogeneity. We found that a healthier lifestyle was associated with a lower risk of GC regardless of the family history of various cancer types, which was consistent with the finding of Jin and his colleagues (21). Compared with the polygenic risk score, which was used in Jin's study (21), family history was widely used in large-scale population screening programs for its convenience and cheapness. Our results will provide a solid foundation for the development of behavior guidance for GC high-risk groups in practical work.

There were some limitations in our study. First, all the information was collected through questionnaire interviews, and the recall bias was unavoidable. For majority of cases, we conducted interviews before cases knew their diagnoses, which might reduce the recall bias to a certain extent. In addition, we also conducted sensitivity analysis after excluding cases from the local Cancer Registry (interviews were conducted after they knew their diagnoses), and the results remained almost unchanged. This alleviated the concern of recall bias to some extent. Second, based on the case-control study design, the causal relationship between exposure and disease cannot be determined. Third, because the food storage method reflected the carcinogens that might be produced by the food (such as nitrites), and physical activity variables have not been collected in our study, our study did not include these two variables. Fourth, there may be potential biases in the selection of cases and controls. However, in our study, the response rates of both the case and the control were high, and there was no statistical difference in age and gender distribution between non-responders and responders, which can dispel this doubt to a certain extent.

In summary, based on this population-based case-control study conducted in Taixing, we confirmed the close relationship between the family history of cancer, especially the family history of early-onset cancer and the risk of GC. We also found that regardless of cancer family history, a good lifestyle was associated with reduced GC risk. The results of our study provide a solid foundation for the development of the behavior guidance for GC high-risk groups.

## Data Availability Statement

The raw data supporting the conclusions of this article will be made available by the authors, without undue reservation.

## Ethics Statement

The studies involving human participants were reviewed and approved by the Ethics Committee of School of Life Sciences, Fudan University, China (date: 19 February 2009), the Ethics Committee of Qilu Hospital of Shandong University, China (date: 8 March 2010), and the Stockholm Ethical Vetting Board, Sweden (2018/357-31). The patients/participants provided their written informed consent to participate in this study.

## Author Contributions

Study supervision was performed by ML. The first draft of the manuscript was written by JM and YN. All authors contributed to the study conception and design, acquisition of data, analysis and interpretation of data, critical revision of the manuscript for important intellectual content, read, approved the final manuscript, and commented on previous versions of the manuscript.

## Funding

This study was supported by the National Natural Science Foundation of China (81573229, 81973116, and 82173591) and National Key Research and Development Program of China (2017YFC0907003). WY was also supported by a grant from the European Research Council (Consolidator Grant No.: 682663).

## Conflict of Interest

The authors declare that the research was conducted in the absence of any commercial or financial relationships that could be construed as a potential conflict of interest.

## Publisher's Note

All claims expressed in this article are solely those of the authors and do not necessarily represent those of their affiliated organizations, or those of the publisher, the editors and the reviewers. Any product that may be evaluated in this article, or claim that may be made by its manufacturer, is not guaranteed or endorsed by the publisher.
