# Supplementary material for: Healthy Lifestyle Factors, Cancer Family History, and Gastric Cancer Risk: A Population-Based Case-Control Study in China
Source: Front Nutr. 2021 Dec 22;8:774530. doi: 10.3389/fnut.2021.774530 (PMC8727865; doi:10.3389/fnut.2021.774530)
Supplement: Supplementary file 1 [file Data_Sheet_1.docx]

**Healthy Lifestyle Factors, Cancer Family History, and Gastric Cancer Risk: A Population-Based Case-Control Study in China**

Jinyu Man^1,2†^, Yingchun Ni^1†^, Xiaorong Yang^2,3^, Tongchao Zhang^1,2^, Ziyu Yuan^4,5^, Hui Chen^2,3^, Xingdong Chen^4,5,6^, Ming Lu^1,2,3,5^ * and Weimin Ye^5,7,8^

†These authors contributed to the work equally and should be regarded as co-first authors.

****Correspondence author***

Ming Lu at Clinical Epidemiology Unit, Qilu Hospital of Shandong University, 107 Wenhuaxi Road, Jinan, Shandong 250012, China. Tel: +86-531-82169051; Fax: +86-531-86927544; Email: [lvming@sdu.edu.cn](mailto:lvming@sdu.edu.cn)

**5 supplementary tables and 1 supplementary figure.**

**List of supporting information**

**Table S1. The association between food storage methods and risk of gastric cancer**

^1^ Model 1 adjusted for age and sex.

^2^ Model 2 adjusted for sex, age, education, marriage, family size, *H. pylori*, and family wealth score.

^3^ Model 3 adjusted for sex, age, education, marriage, family size, *H. pylori*, family wealth score, family history of gastric cancer, smoking, drinking, toothbrushing, and body shape.

Significant ORs with 95% CIs are indicated in bold.

**Table S2. The cigarette smoking status of cases and controls by gender**

The calculation of *P* value was based on chi-squared test (two-sided).

**Table S3. The association between lifestyle factors, lifestyle score and the risk of gastric cancer***

*Excluding cases from the local cancer registry

^1^ Model 1 adjusted for age and sex.

^2^ Model 2 adjusted for sex, age, education, marriage, family size, *H. pylori*, and family wealth score.

^3^ Model 3 adjusted for sex, age, education, marriage, family size, *H. pylori*, family wealth score, and family history of gastric cancer. The variables for calculating the lifestyle score were adjusted to each other in model 3.

Significant ORs with 95% CIs are indicated in bold.

**Table S4. PAFs according to individual and combined lifestyle factors and the risk of gastric cancer***

*Excluding cases from the local cancer registry

PAF: Population attributable fractions

Sex, age, education, marriage, family size, *H. pylori*, family history of gastric cancer, and family wealth score. The variables for calculating the lifestyle score were adjusted to each other.

Significant PAFs with 95% CIs are indicated in bold.

**Table S5. Association between lifestyle score and risk of gastric cancer stratified by family history of malignancy***

*Excluding cases from the local cancer registry

^1^ Model 1 adjusted for age and sex.

^2^ Model 2 adjusted for sex, age, education, marriage, family size, *H. pylori*, and family wealth score.

Significant ORs with 95% CIs are indicated in bold.

**Figure S1. Revised Stunkard's Figure Rating Scale in male (top) and female (bottom)**

Table S1. The association between food storage methods and risk of gastric cancer

| Characteristic | Controls  N=1928 | Cases  N=870 | Gastric cancer | | |
| --- | --- | --- | --- | --- | --- |
|  |  |  | OR  (95% CI)^1^ | OR  (95% CI)^2^ | OR  (95% CI)^3^ |
| Airtight containers |  |  |  |  |  |
| No | 1469  (76.2) | 758  (87.1) | Ref. | Ref. | Ref. |
| Yes | 459  (23.8) | 112  (12.9) | **0.48**  **(0.38,0.60)** | **0.50**  **(0.40,0.63)** | **0.53**  **(0.42,0.67)** |
| Low temperature |  |  |  |  |  |
| No | 1405  (72.9) | 707  (81.3) | Ref. | Ref. | Ref. |
| Yes | 523  (27.1) | 163  (18.7) | **0.63**  **(0.52,0.77)** | **0.67**  **(0.54,0.83)** | **0.70**  **(0.56,0.87)** |

^1^ Model 1 adjusted for age and sex.

^2^ Model 2 adjusted for sex, age, education, marriage, family size, *H. pylori*, and family wealth score.

^3^ Model 3 adjusted for sex, age, education, marriage, family size, *H. pylori*, family wealth score, family history of gastric cancer, smoking, drinking, toothbrushing, and body shape.

Significant ORs with 95% CIs are indicated in bold.

Table S2. The cigarette smoking status of cases and controls by gender

| Characteristics | Total | Controls | Cases | *P* value |
| --- | --- | --- | --- | --- |
| Female |  |  |  | 0.603 |
| Current smoker or former smoker | 33(3.9) | 22(3.7) | 11(4.4) |  |
| Never smoke | 817(96.1) | 579(96.3) | 238(95.6) |  |
| Male |  |  |  | 0.370 |
| Current smoker or former smoker | 1529(78.5) | 1034(77.9) | 495(79.7) |  |
| Never smoke | 419(21.5) | 293(22.1) | 126(20.3) |  |

The calculation of *P* value was based on chi-squared test (two-sided).

Table S3. The association between lifestyle factors, lifestyle score and the risk of gastric cancer*

| Lifestyle factor | Points | Description | Controls  N=1928 | Case  N=870 | Gastric cancer | | |
| --- | --- | --- | --- | --- | --- | --- | --- |
|  |  |  |  |  | OR(95% CI)^1^ | OR(95% CI)^2^ | OR(95% CI)^3^ |
| Body shape | 0 | Unhealthy shape: shape 1/2/5/6/7/8/9 | 672(34.9) | 330(41.8) | Ref. | Ref. | Ref. |
|  | 1 | Healthy shape: shape 3/4 | 1256(65.1) | 460(58.2) | **0.75(0.64,0.89)** | **0.75(0.63,0.90)** | **0.73(0.61,0.87)** |
| Smoking | 0 | Current smoker or former smoker | 1056(54.8) | 464(58.7) | Ref. | Ref. | Ref. |
|  | 1 | Never smoking | 872(45.2) | 326(41.3) | 0.84(0.67,1.06) | 0.86(0.68,1.09) | 0.90(0.71,1.16) |
| Alcohol drinking | 0 | Former or current drinker | 788(48.9) | 369(46.7) | Ref. | Ref. | Ref. |
|  | 1 | Never drinking | 1140(59.1) | 421(53.3) | **0.77(0.63,0.93)** | **0.76(0.62,0.92)** | **0.77(0.63,0.95)** |
| Tooth  brushing | 0 | <=1/day | 1268(65.8) | 660(83.5) | Ref. | Ref. | Ref. |
|  | 1 | >=2/day | 660(34.2) | 130(16.5) | **0.39(0.32,0.49)** | **0.41(0.33,0.51)** | **0.43(0.35,0.54)** |
| Food storage | 0 | Not refrigerated or not sealed | 1310(67.9) | 640(81.0) | Ref. | Ref. | Ref. |
|  | 1 | Refrigerated or sealed | 618(32.1) | 150(19.0) | **0.51(0.42,0.62)** | **0.52(0.42,0.65)** | **0.54(0.43,0.69)** |
| Lifestyle score | 0 |  | 98(5.1) | 88(11.1) | Ref. | Ref. | - |
|  | 1 |  | 369(19.1) | 216(27.3) | **0.63(0.45,0.89)** | **0.62(0.44,0.88)** | **0.60(0.43,0.85)** |
|  | 2 |  | 587(30.4) | 249(31.5) | **0.43(0.31,0.60)** | **0.43(0.31,0.61)** | **0.42(0.30,0.58)** |
|  | 3 |  | 562(29.1) | 181(22.9) | **0.29(0.20,0.41)** | **0.29(0.20,0.42)** | **0.28(0.19,0.40)** |
|  | 4 |  | 243(12.6) | 50(6.3) | **0.18(0.12,0.28)** | **0.19(0.12,0.30)** | **0.19(0.12,0.29)** |
|  | 5 |  | 69(3.6) | 6(0.8) | **0.08(0.03,0.19)** | **0.08(0.03,0.21)** | **0.08(0.03,0.20)** |
| *P* for trend | |  |  |  | **<0.001** | **<0.001** | **<0.001** |

*Excluding cases from the local cancer registry

^1^ Model 1 adjusted for age and sex.

^2^ Model 2 adjusted for sex, age, education, marriage, family size, *H. pylori*, and family wealth score.

^3^ Model 3 adjusted for sex, age, education, marriage, family size, *H. pylori*, family wealth score, and family history of gastric cancer. The variables for calculating the lifestyle score were adjusted to each other in model 3.

Significant ORs with 95% CIs are indicated in bold.

Table S4. PAFs according to individual and combined lifestyle factors and the risk of gastric cancer*

| Lifestyle factor | Gastric cancer | |
| --- | --- | --- |
|  | Proportion of cases with risk factor | PAF (95% CI) |
| Unhealthy body shape | 330(41.8%) | **11%(5%,16%)** |
| Smoking | 464(58.7%) | 6%(-10%,18%) |
| Alcohol drinking | 369(46.7%) | **10%(3%,17%)** |
| Tooth brushing <=1 per day | 660(83.5%) | **48%(39%,55%)** |
| Bad food storage method | 640(81.0%) | **37%(26%,46%)** |
| Lifestyle score<3 | 553(70.0%) | **36%(28%,43%)** |

*Excluding cases from the local cancer registry

PAF: Population attributable fractions

Sex, age, education, marriage, family size, *H. pylori*, family history of gastric cancer, and family wealth score. The variables for calculating the lifestyle score were adjusted to each other.

Significant PAFs with 95% CIs are indicated in bold.

Table S5. Association between lifestyle score and risk of gastric cancer stratified by family history of malignancy*

| Family history of malignancy | Lifestyle score | Controls  N=1928 | Cases  N=790 | Gastric cancer | |
| --- | --- | --- | --- | --- | --- |
|  |  |  |  | OR  (95% CI)^1^ | OR  (95% CI)^2^ |
| Gastric cancer | <=2 | 110  (5.7) | 101  (12.8) | Ref. | Ref. |
|  | >=3 | 102  (5.3) | 43  (5.4) | **0.49**  **(0.30,0.82)** | **0.48**  **(0.28,0.82)** |
| Other digestive system cancer | <=2 | 232  (12.0) | 121  (15.3) | Ref. | Ref. |
|  | >=3 | 203  (10.5) | 71  (9.0) | 0.68  (0.45,1.01) | 0.73  (0.48,1.12) |
| Other cancer | <=2 | 135  (7.0) | 70  (8.9) | Ref. | Ref. |
|  | >=3 | 91  (4.7) | 23  (2.9) | **0.33**  **(0.18,0.63)** | **0.37**  **(0.18,0.75)** |
| No family history | <=2 | 577  (30.0) | 261  (33.0) | Ref. | Ref. |
|  | >=3 | 478  (24.8) | 100  (12.7) | **0.40**  **(0.30,0.54)** | **0.41**  **(0.30,0.55)** |

*Excluding cases from the local cancer registry

^1^ Model 1 adjusted for age and sex.

^2^ Model 2 adjusted for sex, age, education, marriage, family size, *H. pylori*, and family wealth score.

Significant ORs with 95% CIs are indicated in bold.

**
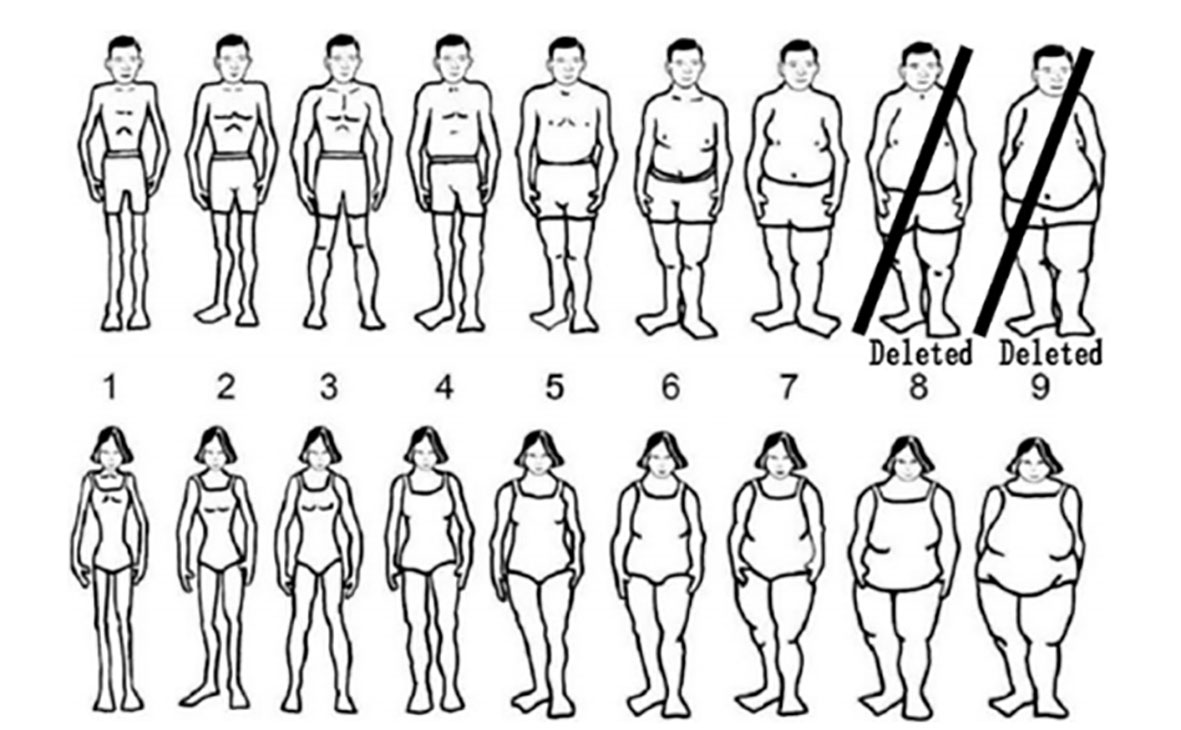
Figure S1. Revised Stunkard's Figure Rating Scale in male (top) and female (bottom)**
